# Comparison of tension-free transvaginal tape and transobturator tape in terms of urinary incontinence and quality of life among Turkish women^*^

**DOI:** 10.1590/1806-9282.20250355

**Published:** 2025-10-17

**Authors:** Pakize Özge Karkin, Gözde Sezer, Nehir Pişkin, Demet Yilmaz

**Affiliations:** 1 University of Health Sciences, Istanbul Kanuni Sultan Süleyman Training and Research Hospital – Istanbul, Turkey.; 2 Celal Bayar University – Manisa, Turkey.; 3 Sisli-16 Family Medicine Health Center – Istanbul, Turkey.

**Keywords:** Quality of life, Urinary incontinence, Suburethral slings

## Abstract

**OBJECTIVE:**

Urinary incontinence is more common in women than men; additionally, prevalence rises in elderly women. This study was conducted to compare the 10-year results of tension-free transvaginal tape and transobturator tape operations in terms of urinary incontinence and quality of life in women.

**METHODS:**

Women who underwent transvaginal tape and transobturator tape operations in the urogynecology department at Istanbul Kanuni Sultan Süleyman Training and Research Hospital between January and December 2013 were retrospectively screened. The Urogenital Distress Inventory-6 and King's Health Questionnaire were applied to volunteers who could be reached via phone between January 2023 and July 2023 to evaluate urinary incontinence and quality of life.

**RESULTS:**

Of the 117 volunteers who were included in the study, 28 underwent transvaginal tape and 89 underwent transobturator tape operations. A statistically significant difference in favor of transvaginal tape was found between the two groups in terms of relief from postoperative stress urinary incontinence and pelvic pain (p<0.05). When transvaginal tape and transobturator tape groups were compared according to Urogenital Distress Inventory-6 and King's Health Questionnaire, no statistically significant difference was found (p>0.05). Although it is not statistically significant, it can be thought that transvaginal tape is superior than transobturator tape in terms of quality of life, by patient satisfaction with minimal clinically important difference.

**CONCLUSION:**

Although there is no long-term statistical difference in terms of quality of life between transvaginal tape and transobturator tape surgery, the transvaginal tape procedure was more successful in the long term, subjectively, in terms of stress urinary incontinence and postoperative pelvic pain among women.

## INTRODUCTION

Urinary incontinence (UI) is a common involuntary urinary leakage problem that affects mostly elderly people and is more common in women. While its prevalence is 20% under the age of 20, it rises to 50% in older ages^1,2^.

UI is defined by the International Continence Society (ICS) and the International Urogynecological Association (IUGA) as involuntary urinary leakage that causes social problems^3^. UI is defined under three common subtypes as stress, urgency, and mixed UI^4,5^. Stress urinary incontinence (SUI) is the most common subtype of UI^1^. Due to urethral hypermobility, SUI can be seen during coughing, sneezing, or other physical activities which cause increased intra-abdominal pressure. Urgency urinary incontinence (UUI) is urination before reaching the toilet with an urgent need for micturition, which occurs due to excessive activity of the detrusor muscles. The coexistence of stress incontinence and urge incontinence is called mixed-type UI. Risk factors for UI include advanced age, short urethral neck in women, hypoestrogenemia, pregnancy, birth trauma, recurrent resistant urinary infections, obesity, gynecological operations, diabetes, peripheral vascular insufficiency, neurological diseases, and congestive heart failure^6^. Although UI is seen at all ages and genders, it is a common problem, especially in elderly women. It affects one-third of women and about one-fifth of men over the age of 60 in the world^4,5^.

UI negatively affects physical, social, and sexual life^7^.

UI treatment ranges from lifestyle changes to medical and surgical methods^8^. Determining the type of incontinence may affect the success of the treatment, especially in cases where surgical treatment is considered. Mid-urethral sling procedures, which have gained popularity in the last 20 years, have become more prominent, replacing urethral suspension surgeries. Tension-free transvaginal tape (TVT) and transobturator tape (TOT) are mid-urethral sling procedures^2,9^.

Since investigating the long-term success of UI surgeries and the quality of life (QoL) of patients after the operation is important, our study aims to compare the 10-year results of TVT and TOT operations in terms of UI and QoL.

## METHODS

### Study population

**Figure 1:**
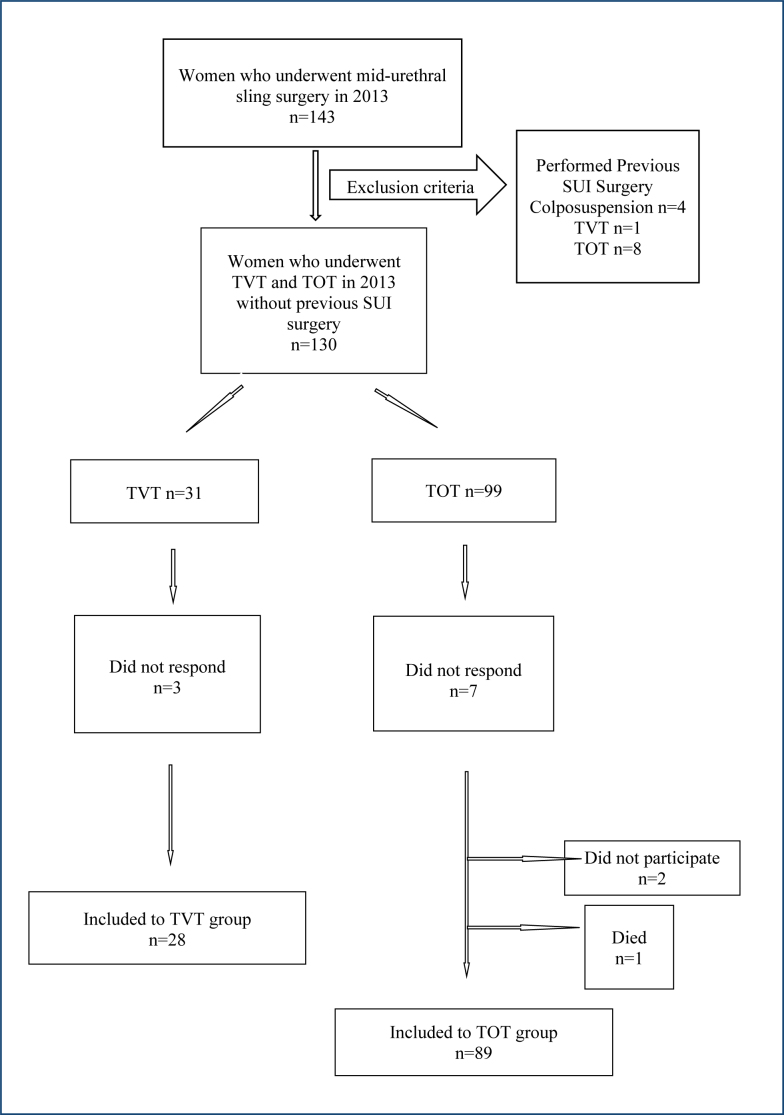
Flow chart of the study population.

Women who had been hospitalized in the urogynecology department of Istanbul Kanuni Sultan Süleyman Training and Research Hospital and had undergone TVT and TOT operations between the dates 01.01.2013 and 31.12.2013 were retrospectively screened from the hospital's health repository. Previously performed SUI surgeries (colposuspension, autologous rectus fascial sling, mid-urethral slings) and incomplete or missing operation data were exclusion criteria. The population included in the study were those undergoing their first SUI surgery. Between January 2023 and July 2023, appropriate candidates were called, informed, and asked to volunteer for the study. One hundred and seventeen volunteers are accepted to be included (Figure 1). Sample size for frequency in a population was calculated with the Epi info programme. Study population size is above the 95% confidence level. The estimated effect size was Cohen's d=0.54. A post-hoc power analysis was conducted using the G*Power 3.1 software. With an alpha level of 0.05 and an independent samples t-test, the statistical power (1-β) of the study was calculated to be approximately 83%, indicating a moderate level of statistical power for two different midurethral sling comparisons.

### Surgical techniques

The study population who were suffering from SUI, with or without pelvic organ prolapse (POP), underwent mid-urethral sling procedures with or without concomitant POP surgery. All mid-urethral sling procedures were performed by two experienced surgeons who had been responsible for the urogynecology department since 2005. The polypropylene mesh was used. Perioperative 30° cystoscopy was performed after passing the trocars in TVT. The retropubic route was inside-out. No cystoscopy data was found during the surgery for TOT. The transobturator route was outside-in.

### Questionnaires

Urogenital Distress Inventory (UDI-6) and King's Health Questionnaire (KHQ) were applied to evaluate UI and QoL, and questions including sociodemographic characteristics and obstetric characteristics were asked.

UDI-6 assesses life quality and symptom distress in women suffering from UI.

The UDI-6 includes three sub-dimensions: irritative symptoms, stress symptoms, and obstructive/disturbing or voiding symptoms, and consists of six questions. In the scoring of the scale, there are options from 0 to 3. A minimum of 0 to a maximum of 18 points can be obtained from UDI-6. A rise in the score indicates a deterioration in the QoL^10^.

The KHQ is an assessment of health-related QoL related to a specific condition (bladder problems). Twenty-one items about urinary tract symptoms yield scores in nine domains (general health perception, incontinence impact, role limitations, physical limitations, social limitations, personal relationships, emotions, sleep/energy, and severity of symptoms). Each item is rated using a 4- or 5-point Likert scale. Domain scores range from 0 (best) to 100 (worst). There are two single-item domains (general health perceptions and incontinence impact), and the severity of symptoms domain is scored using a scale from 0 (best) to 30 (worst). As the score increases, the QoL decreases^11^.

### Statistical analysis

Statistical analysis was performed with Statistical Package for Social Sciences (IBM SPSS Statistics for Windows, Version 21.0. Armonk, NY: IBM Corp.). In the analysis of data, numerical and percentage distribution, mean, standard deviation, the Kolmogorov-Smirnov test for comparison between groups, and the t-test for independent groups were used when normal distribution was achieved. The chi-square test was used in the analysis of categorical variables. The p<0.05 was considered statistically significant.

### Ethics

The approval for the study was obtained from the ethics committee of Istanbul Kanuni Sultan Süleyman Training and Research Hospital (number 2022.12.238). The study was conducted in accordance with the Declaration of Helsinki.

## RESULTS

Sociodemographic characteristics of the patients were analyzed among the patients who had undergone TVT and TOT 10 years ago in terms of age, body mass index (BMI), menopause, hormone replacement therapy, UI problem, and sudden incontinence when rushing to the toilet, POP (uterine prolapse/cystocele/rectocele), and problems during sexual intercourse. No significant difference was found statistically (p>0.05) (Table 1).

**Table 1:** Sociodemographic characteristics of the patients.

	TVT n=28	TOT n=89	p
Age	63.39	63.92	0.959
BMI (kg/m^2^)	26.89	26.79	0.554
Menopause	19 (67.9%)	69 (77.5%)	0.418
Hormone replacement therapy	None 22 (78.6%)	None 59 (66.3%)	0.160
Urinary incontinence problem	None 18 (64.3%)	None 45 (50.6%)	0.146
Urinary incontinence in daily activities such as coughing	Exists 8 (28.6%)	Exists 48 (53.9%)	0.016
Urgent need and involuntary leakage before going to the toilet	None 15 (53.6%)	None 48 (53.9%)	0.572
POP	None 16 (57.1%)	None 56 (62.9%)	0.370
Problems during sexual intercourse	None 14 (50.0%)	None 39 (43.8%)	0.537
Postoperative pelvic pain	Exists 16 (57.1%)	Exists 68 (76.4%)	0.044

BMI: body mass index, POP: pelvic organ prolapse; TVT: transvaginal tape; TOT: transobturator tape. Data is given as n (%) and mean.

The effects of age, BMI, menopause, hormone therapy, and pregnancy variables on TVT and TOT surgical outcomes are not statistically significant (p>0.05). Parity was the only statistically significant variable (p<0.05, p<0.05) (Table 2).

**Table 2:** Multivariate regression of patient variables.

	TVT						TOT					
	B Coeff.	Standard error	p-value	95%CI (lower–upper)		VIF	B Coeff.	Standard error	p-value	95%CI (lower–upper)		VIF
Age	0.017	0.019	0.363	-0.021	0.056	3,976	-0.014	0.009	0.110	-0.031	0.003	1,976
BMI	0.030	0.108	0.786	-0.195	0.255	1,169	-0.134	0.101	0.190	-0.335	0.068	1,374
Menopause	0.075	0.184	0.686	-0.458	0.307	3,684	-0.153	0.094	0.107	-0.339	0.034	1,441
HRT	0.152	0.204	0.465	-0.273	0.576	1,427	-0.160	0.100	0.115	-0.360	0.040	1,084
Delivery	0.238	0.092	0.045	0.009	0.429	7,202	0.104	0.049	0.037	0.007	0.202	4,733
Pregnancy	-0.078	0.066	0.211	-0.280	-0.028	6,988	-0.060	0.049	0.230	-0.038	0.157	3,204

Coeff.: coefficient; HRT: hormone replacement therapy; BMI: body mass index; TVT: transvaginal tape; TOT: transobturator tape; CI: confidence interval; VIF: variance inflation factor.

Table 3 shows the complications.

**Table 3:** Mid-urethral sling surgery complications.

	TVT n=28					TOT n=89				
		n	%	CCS	Re-in		n	%	CCS	Re-in
Intraoperative	Bladder perforation	2	7.1	None	None	Vaginal sulcus perforation	1	1.1	CA	None
Postoperative	Dyspareunia	1	3.6	MF	None	Dyspareunia	1	1.1	CA	None
	Urinary retention	1	3.6	Note	Tapecut	Mesh erosion	3	3.4	CA	2 res.
						Granuloma in bladder¶	1	1.1	CA	Exc.
						RUTI**	1		CA	None
						OAB**	1		CA	bot.2
Overall re-intervention rate for all complications		n	%				n	%		
		1	3.6				5	5.6		

Data is given as number and ratio; TVT: transvaginal tape; TOT: transobturator tape; RUTI: recurrent urinary tract infection; OAB: overactive bladder; CCS: concomitant surgery; Re-in: reintervention; MF: Manchester-Fothergill operation; CA: colporrhaphy anterior; res.: partial resection; exc.: excision; bot.: intravesical botox application, bot2 means intravesical botox application twice. Re-intervention rate is given by the number of applications.

¶ Secondary complications occurred after this complication.

** Means secondary complications in accordance with TOT surgery.

Intraoperative complications were more common during TVT than TOT. Two cases (7.1%) of bladder perforation while inserting trocars were seen in the TVT group, immediate removal and reinsertion were performed without complication.

One case (1.1%) of vaginal sulcus perforation was found in TOT and mucosal defect repaired intraoperatively, no following complication was seen afterward.

Postoperative TVT complications were reported as: 1 (3.6%) seeking treatment for dyspareunia who underwent concomitant Manchester-Fothergill operation, 1 (3.6%) urinary retention, which was treated by cutting the tape after 1 week from surgery.

Postoperative TOT complications were noted as: 3 (3.4%) mesh erosion, 1 treated with local topical estrogen, 2 treated with partial resection. One case (1.1%) of dyspareunia. One case (1.1%) of granuloma-like tissue in the bladder mucosa after 5 months postoperatively, treated with cystoscopic resection. The patient who received granuloma resection, her complaints continued with recurrent urinary tract infections and ended up with a new onset of overactive bladder in the last 5-year period. She received intravesical Botox treatment twice. All postoperative TOT complications occurred with concomitant anterior colporrhaphy. The overall re-intervention rates in terms of complications accordance to TVT and TOT were found to be 3.6 and 5.6%, respectively.

The study population were asked about the presence of urinary leakage in daily activities such as coughing, it was determined that 53.9% of the patients who underwent TOT surgery had UI, and 71.4% of those who had TVT did not. A statistically significance was found between TVT and TOT according to SUI during postoperative 10th year (p<0.05) (Table 1).

As the postoperative pelvic pain, which intermittently continued during 10 years after the operation, was compared, it was found that 76.4% of the TOT group had pain and while 57.1% of the TVT group had pain. There was a statistically significant difference in postoperative pelvic pain between the two procedures (p<0.05) (Table 1).

The comparison of UDI-6 between TVT and TOT shows no difference statistically in terms of long-term QoL (p>0.05) (Table 4).

**Table 4:** Comparison of transvaginal tape and transobturator tape according to Urogenital Distress Inventory-6 and King's Health Questionnaire; minimal clinically important difference between mid-urethral sling surgeries in terms of quality of life.

UDI-6	TVT n=28	TOT n=89	p
Frequent urination	2.39±0.87	2.41±0.75	0.901
Urine leakage related to urgency	2.25±0.88	2.31±0.82	0.734
Urine leakage related to physical activity (walking, running, laughing, sneezing, coughing)	2.28±0.97	2.21±0.87	0.728
Small amounts of urine leakage (drops)	2.21±0.91	2.07±0.95	0.503
Difficulty emptying your bladder or difficulty urinating	2.14±0.80	2.03±0.92	0.548
Pain or discomfort in your lower abdominal, pelvic, or genital area	2.25±0.88	2.12±0.90	0.516
UDI-6 total scores	13.54±5.02	13.18±4.83	0.743
MCID	8.69	8.68	
**KHQ**			
General health perceptions Min–max. score (25–75)	41.07±16.96	41.29±16.89	0.952
Incontinence impact Min–max. score (0–100)	58.32±28.14	55.42±22.99	0.623
Role limitations Min–max. score (0–100)	41.66±35.85	38.57±29.36	0.646
Physical limitations Min–max. score (0–100)	42.85±34.96	38.38±31.22	0.549
Social limitations Min–max. score (0–100)	42.85±32.66	37.45±29.74	0.440
Personal relationships Min–max. score (0–100)	39.28±36.06	29.96±29.42	0.169
Emotions Min–max. score (0–100)	37.69±27.76	32.83±27.05	0.420
Sleep/energy Min–max. score (0–100)	39.87±32.18	32.95±24.61	0.232
Severity measures Min–max. score (0–100)	54.51±26.17	53.47±21.50	0.850
Symptom severity scale Min–max. score (0–30)	18.14±5.92	16.19±6.48	0.144
KHQ total scores	49.64±15.93	46.89±14.00	0.417
MCID	27.28	24.67	

UDI-6: Urogenital Distress Inventory-6; KHQ: King's Health Questionnaire; MCID: minimal clinically important difference; TVT: transvaginal tape; TOT: transobturator tape. Data is given as mean±SD and exact number.

In terms of KHQ, there is no statistical significance for long-term QoL between TVT and TOT surgeries in all domains (p>0.05) (Table 4).

Overall results for QoL on UDI-6 and KHQ are not statistically different for TVT and TOT patients at long-term (p>0.05). Although there was no statistically significant difference and higher numerical scores, as we focus on minimal clinically important difference (MCID), in terms of KHQ, TVT increases the QoL of the SUI-suffering women better than TOT (Table 4).

## DISCUSSION

Although UI is a problem with increasing prevalence by age in society, recently, it is seen as a problem that impairs the QoL among patients, and the search of need for treatment has increased. Minimally invasive techniques for treating SUI have gained popularity in recent years since they were first described, but there is still not enough data in the literature regarding the 10-year results of mid-urethral sling operations performed in the treatment of SUI.

After TVT was first described in 1996 and had been commonly used for years, complications were reported; therefore, TOT surgery was described in search of finding a safe route for sling operations, since minimally invasive operations are blind procedures. However, new and different complications were reported later. In a cadaver study evaluating the distances to important anatomical structures that may cause complications in midurethral sling surgeries, it was reported that as BMI increased, the distance to these structures decreased, but this was not statistically significant^12^.

Similarly, in our study, BMI did not affect TVT and TOT results. Thus, it is thought that BMI is not associated with complications.

In a meta-analysis comparing TVT and TOT operations in terms of evaluation of pain and QoL, VAS and UDI-6 scores were higher in the TOT group than in the TVT group, but without statistical significance^13^. Similar results for pain but contrary results for UDI-6 were found in our study, which showed the TVT group had higher numerical scores for QoL but less pain than TOT after 10-year follow-up, yet the difference for QoL was not statistically significant. Indeed, our study states MCID was the same for UDI-6 in terms of QoL. Additionally, in the same meta-analysis, there was no significant difference in cure rate and satisfaction rate between the groups^13^. After a 4-year comparison of two procedures in another study, no significant difference was found between the procedures in terms of continence results and QoL. But the complication rate was significantly higher with the TVT procedure when compared to the TOT procedure^14^. Our study states approximately similar complication rates postoperatively between the two mid-urethral sling surgeries. Even though dyspareunia seems to be at a higher rate in the TVT group, this can be thought to be because of the concomitant surgery of Manchester-Fothergill operation. In other words, this dyspareunia may have also occurred due to the cervical amputation, not because of TVT. Although there were higher intraoperative complication rates in TVT, these were treated during surgery and did not cause long-term complications. A randomized trial reports higher perioperative complications for TVT than for the inside-out approach of TOT^15^. A study that discusses the complication comparison, more mesh erosion and groin pain were reported in TOT than in TVT among short-term complications, while the reoperation rate was found to be higher in TVT than in TOT at 16 months^16^. On the other hand, our long-term result study showed that the overall re-intervention rate is higher for TOT than TVT. Surgeons may assume that the TOT route is safer and simpler than TVT, but because of the longer path of the mesh in the vagina, there appears to be a greater risk of mesh erosion and related long-term complications and re-intervention rate, according to our data for the transobturator procedure. Indeed, as found in this study, concomitant anterior colporrhaphy may be a risk-increasing factor for mesh erosion or displacement and long-term mesh-related complications. This may have been due to the fact that concomitant surgeries were performed with a single vaginal incision. Paying attention to the incision may prevent complications.

In randomized trials comparing two procedures, pelvic pain was greater in TOT in 1-year follow-up, but at 5 years, pain in the groin and pelvic pain were reported higher in the TVT group^17,18^. By contrast, our 10-year follow-up states pelvic pain is greater in patients who have undergone TOT. In a supportive way, a systematic review also states that leg and groin pain is more common for inside-out TOT than TVT^19^.

A trial that was comparing 5-year results between two mid-urethral sling procedures revealed no statistical difference in terms of SUI, in parallel with our statistical study results, supported with questionnaire^18^. Although there is no statistical significance, according to patient satisfaction, we found that MCID shows better QoL for continence for TVT.

Yet, results differ in urge UI; in 5-year follow-up, urge incontinence was statistically higher in that trial's TVT group^18^. Our study, which shows 10-year results, revealed no difference according to urge UI between TVT and TOT patients. The UDI-6 score difference was marginal between TVT and TOT in the 5-year follow-up^18^. Despite the total numerical scores being slightly higher in the TVT group after 10-year follow-up, , our UDI-6 and KHQ results were not statistically different between the two groups. In spite of higher numerical scores, MCID states better long-term QoL for continence among TVT-performed patients than the TOT group.

Some conflicting results between studies can generally be explained by differences in follow-up periods and methods.

Although surgeons find the TOT route safer than TVT, as a consequence of our study data, TOT is associated with more procedure-related pain and less SUI control than TVT in the long period^20^.

Especially by recognizing the perioperative complications and ignoring the prolongation of operation time for the retropubic procedure, shifting the surgical choice toward TVT, between mid-urethral sling surgeries, may be more beneficial in order to reduce operation-related pelvic pain and improve the QoL.

The major strength of this study is that it obtained the long-term results. Another important aspect is not only focusing on statistical results but also caring about the patient's benefit and satisfaction by a MCID. Yet, several limitations are worth pointing out, which are difficulty reaching patients after a 10-year period, having a small TVT population, and being a single-center study.

## CONCLUSION

As a result, although there was no long-term statistical difference in the QoL due to UI among patients who underwent TVT and TOT surgeries; subjectively, the TVT procedure seemed to be more successful with regard to SUI and postoperative pelvic pain in long term.

## Data Availability

The datasets generated and/or analyzed during the current study are available from the corresponding author upon reasonable request.
